# N6-methyladenosine methylation: a novel key to unlocking mental disorders

**DOI:** 10.1093/ijnp/pyaf044

**Published:** 2025-07-01

**Authors:** Yinuo Wang, Xiaobing Li, Min Liu, Xiaoxu Xu, Yue Ma, Yang Luo, Yue Wang

**Affiliations:** Medical Research Center, Jinan Maternity and Child Care Hospital Affiliated to Shandong First Medical University, Jinan, Shandong, China; Department of Human Anatomy, School of Clinical and Basic Medical Sciences, Shandong First Medical University & Shandong Academy of Medical Sciences, Jinan, Shandong, China; Medical Research Center, Jinan Maternity and Child Care Hospital Affiliated to Shandong First Medical University, Jinan, Shandong, China; Department of Human Anatomy, School of Clinical and Basic Medical Sciences, Shandong First Medical University & Shandong Academy of Medical Sciences, Jinan, Shandong, China; Medical Research Center, Jinan Maternity and Child Care Hospital Affiliated to Shandong First Medical University, Jinan, Shandong, China; Department of Human Anatomy, School of Clinical and Basic Medical Sciences, Shandong First Medical University & Shandong Academy of Medical Sciences, Jinan, Shandong, China; Department of Human Anatomy, School of Clinical and Basic Medical Sciences, Shandong First Medical University & Shandong Academy of Medical Sciences, Jinan, Shandong, China; Medical Research Center, Jinan Maternity and Child Care Hospital Affiliated to Shandong First Medical University, Jinan, Shandong, China; Department of Human Anatomy, School of Clinical and Basic Medical Sciences, Shandong First Medical University & Shandong Academy of Medical Sciences, Jinan, Shandong, China; Department of Human Anatomy, School of Clinical and Basic Medical Sciences, Shandong First Medical University & Shandong Academy of Medical Sciences, Jinan, Shandong, China; Medical Research Center, Jinan Maternity and Child Care Hospital Affiliated to Shandong First Medical University, Jinan, Shandong, China; Department of Human Anatomy, School of Clinical and Basic Medical Sciences, Shandong First Medical University & Shandong Academy of Medical Sciences, Jinan, Shandong, China

**Keywords:** m6A methylation, mental disorders, learning and memory, substance addiction

## Abstract

More than 100 types of RNA modifications have been identified in mammalian cells, among which N6-methyladenosine (m6A) is the most prevalent. This reversible and dynamic modification involves methyltransferases, demethylases, and reader proteins. Aberrant expression of m6A-related regulatory proteins in the nervous system significantly impacts neuronal physiology, contributing to mental disorders such as depression, autism spectrum disorder, and schizophrenia. This review summarizes the role of m6A methylation in the pathogenesis of mental disorders and highlights its potential as a biomarker and therapeutic target, providing a comprehensive reference for future research and clinical interventions.

## INTRODUCTION

Epigenetic modifications are heritable changes in gene expression driven by environmental factors or intracellular mechanisms that do not alter the DNA sequence.^1^ These modifications include DNA methylation, RNA methylation, chemical modifications of proteins, non-coding RNA regulation, and so on.^2^ In recent years, advances in molecular biology have significantly deepened our understanding of RNA post-transcriptional modifications. Among these, N6-methyladenosine (m6A), the most abundant of over 160 known RNA modifications, has garnered substantial attention.^3^ N6-methyladenosine marks specific sequences or structures in RNA molecules by adding a methyl group to the nitrogen atom at the sixth position of adenosine.^4^ This modification plays a critical role in regulating RNA stability, splicing, translation, and localization.^5^^,^^6^

Mental illnesses represent a significant global challenge, imposing substantial burdens on individuals, society, and economies.^7^ Current treatments primarily involve pharmacological interventions and psychological therapies.^8^ However, drug efficacy varies greatly due to individual differences, and some patients may often develop drug tolerance or dependency.^9^ Furthermore, psychological treatments are influenced by various external factors easily. These limitations highlight the urgent need for further research into the pathogenesis of mental illnesses and the development of novel therapeutic approaches.

The m6A modification is crucial for the normal development and physiological functioning of the nervous system, including its roles in neurogenesis, neuronal differentiation, synaptic transmission, and the cognitive function.^10^^,^^11^ By regulating the expression of specific transcription factor and their interactions with downstream target genes, m6A modifications help maintain the brain’s structure and function.^12^ So dysregulation of m6A may profoundly impact brain physiology, contributing to the onset of neurological and mental disorders.^13^

Genetic loci regulating m6A levels as quantitative traits (m6A quantitative trait loci, or m6A-QTLs) have been identified in various human tissues, including the brain.^14^ Integration of m6A-QTLs with genome-wide association studies (GWAS) has revealed 184 colocalized loci, among which brain-specific m6A-QTLs are significantly associated with neuroticism, depression, schizophrenia, and anxiety. These findings provide further evidence supporting the involvement of m6A modification in the pathogenesis of mental disorders.

In this review, we summarize the molecular mechanisms underlying m6A methylation, its roles in psychiatric disorders, and its emerging potential as a therapeutic target. To achieve this, we conducted a systematic literature search of studies published before October 2024 using PubMed, Web of Science, and Google Scholar. We included original research articles and review papers investigating m6A methylation in the central nervous system and its association with mental disorders, based on human, animal, or cellular models. Articles were selected for their scientific relevance, methodological rigor, and novelty. Given the high prevalence of certain psychiatric conditions and the extent of related m6A research, this review focuses specifically on depression, autism spectrum disorder (ASD), schizophrenia, and substance addiction.

## THE BIOLOGICAL BASIS OF M6A METHYLATION MODIFICATION

### Dynamic Regulation of m6A Methylation Modification

**Figure 1:**
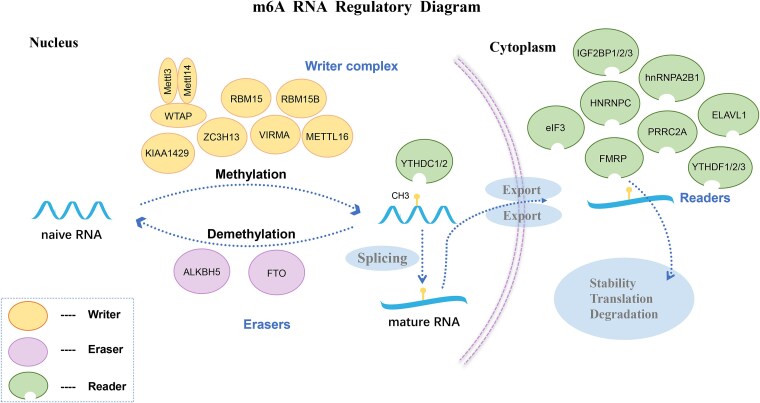
Overview of the m6A RNA modification machinery. N6-methyladenosine (m6A) methylation is catalyzed by a multi-component writer complex comprising METTL3, METTL14, METTL16, WTAP, VIRMA (KIAA1429), RBM15/15B, and ZC3H13. These methyltransferases install m6A modifications on mRNA bases. The modification can be reversed by eraser enzymes such as FTO and ALKBH5, which mediate m6A demethylation. Reader proteins, including YTHDF1/2/3, YTHDC1/2, IGF2BP1/2/3, FMRP, PRRC2A, ELAVL1, eIF3, and HNRNPC/A2B1, recognize and bind to m6A-modified RNA to regulate various post-transcriptional processes, such as mRNA translation, degradation, and microRNA processing.

The m6A methylation modification is a dynamic and reversible process regulated by 3 core groups of factors: methyltransferases (“writers”), demethylases (“erasers”), and reader proteins (“readers”).^15^ Methyltransferases, such as METTL3, METTL14,^16^ WTAP,^17^ KIAA1429,^18^ ZC3H13,^19^ RBM15/15B,^20^ and VIRMA,^21^ are responsible for adding methyl groups to RNA. Demethylases, including ALKBH5^22^ and FTO,^23^ reverse this modification by removing methyl groups, while reader proteins, such as YTHDF1,^24^ YTHDF2,^25^ YTHDF3,^26^ IGF2BP1/2/3,^27^ FMRP,^28^ PRRC2A,^29^ hnRNPA2B1,^30^ eIF3,^31^ YTHDC1,^32^ YTHDC2,^33^ ELAVL1,^34^ and HNRNPC,^35^ recognize and mediate the biological functions of m6A. Together, these factors create a highly dynamic regulatory network that controls RNA methylation (Figure 1). These processes influence critical cellular functions, including self-repair, differentiation, invasion, and apoptosis, and are essential for maintaining cellular homeostasis.^27^^,^^36^

### Biological Functions of m6A Methylation Modification

The biological functions of m6A methylation modification span a wide range of physiological processes, including hematopoietic stem cell differentiation, spermatogenesis, neurogenesis, the cell cycle, and RNA metabolism.^5^^,^^37^^,^^38^ In the nervous system, m6A plays an indispensable role in neurogenesis, brain development, myelin formation, synaptic plasticity, and learning and memory.^34^ Dysregulation of m6A is associated with numerous neurological diseases, such as cerebral ischemia, brain tumors, Alzheimer’s disease (AD), and Parkinson’s disease (PD).^39^ Additionally, m6A modification mediates the body’s response to environmental stress, serving as a molecular bridge between intracellular changes and behavioral abnormalities.^40^ For example, studies on the Chinese Han population have identified ALKBH5, a demethylase, as a risk factor of a stress-related disease, major depressive disorder (MDD).^41^ In animal models of depression induced by chronic restraint stress, decreased expression of another demethylase, FTO, in the hippocampus impairs synaptic plasticity, leading to depressive-like behaviors either.^42^ Notably, overexpression of FTO can reverse these structural and behavioral changes, and antidepressant treatment with fluoxetine has been shown to upregulate hippocampal FTO expression, further demonstrating the role of m6A in depression.^43^

#### The Role of m6A Methylation Modification in Neural Development

The m6A modification is essential for nervous system development. The methyltransferases METTL3 and METTL14 play pivotal roles in neuronal differentiation; their deletion impairs the ability of neural stem cells to differentiate into neurons, disrupting neuronal formation and function.^44^ Similarly, reader proteins like YTHDF2 also play critical roles in this process^45^ Downstream molecules, such as Fragile X Mental Retardation Protein (FMRP) and components of the Wnt/β-catenin signaling pathway, are involved in mediating these effects.^28^^,^^46^

The m6A modification also has effects on oligodendrocyte specialization and myelin formation. By recognizing the GGACU consensus motif in the Olig2-encoding sequence, the reading protein PRRC2A binds and stabilizes Olig2 mRNA in an m6A-dependent mechanism.^29^ This regulation facilitates the specialization and myelination of oligodendrocytes, ultimately influencing their maturation. Additional, abnormalities in METTL14 disrupt the methylation of key transcription factors, further hindering glial development.^47^ In the context of axonal development, m6A modification is indispensable for multiple processes. For example, FTO facilitates axonal elongation in dorsal root ganglion neurons by demethylating Gap43 mRNA.^48^ Similarly, the deletion of YTHDF1 in spinal cord neurons results in axonal guidance defects.^49^

These findings underscore the diverse functions of m6A modification at various stages of nervous system development, including neurogenesis, neuronal differentiation, glial cell maturation, and axonal growth. The dynamic regulation of m6A methylation highlights its critical importance as a fundamental mechanism underlying both normal physiological processes and pathological conditions.

#### The Role of m6A Methylation in Neural Plasticity

Neural plasticity, the remarkable ability of the nervous system to adapt to internal and external environmental changes, underpins advanced neural functions such as cognition and emotion. In neurodegenerative diseases such as AD, dysregulation of m6A is closely linked to synaptic protein dysfunction, impairing synaptic plasticity and contributing to deficits in learning and memory.^50^^,^^51^ Emerging evidence highlights the critical role of m6A methylation in regulating neural plasticity by modulating the expression of plasticity-related genes, thus contributes to the onset and progression of mental disorders.

In the hippocampus, m6A methylation affects neural plasticity by modulating the miR-124-C/EBPα-FTO transcriptional axis, which regulates the expression of neural plasticity-related genes, such as *Nr3c1*, *Creb1*, *Bdnf*, and *Ntrk2*, in the learned helplessness rat model, a key paradigm for studying depression.^52^ In addition, the demethylase ALKBH5 has been implicated in synaptic labeling and synaptic plasticity. By modulating m6A modification levels, ALKBH5 influences synaptic connectivity and function, highlighting its crucial role in synaptic dynamics and plasticity.^53^

Moreover, recent studies suggest that physical exercise can mitigate mental disorders by counteracting chronic stress-induced neural dysfunction.^54^^,^^55^ This protective effect is closely associated with m6A methylation changes in the medial prefrontal cortex (PFC), as well as with the regulation of synaptic plasticity.^56^ Gut microbiota dysbiosis, another consequence of chronic stress, has also been identified as a contributing factor in mood disorders and represents a promising therapeutic target.^57-59^ Notably, m6A modification influences microbial diversity and intestinal barrier integrity. Conversely, alterations in the gut microbiota can affect m6A methylation levels by modulating the recruitment and enzymatic activity of m6A regulatory proteins, thereby participating in the pathogenesis of neuropsychiatric conditions.^60^

In addition to stress factors, exposure to certain chemicals in the environment can also alter the level of m6A modification in neurons, thereby affecting neuroplasticity. For example, exposure to bisphenol A (BPA) results in an increase in m6A levels in hippocampal neurons, along with an upregulation of METTL3 expression. Treatment with STM2457, a selective METTL3 inhibitor, effectively suppresses BPA-induced m6A upregulation and restores normal synaptic transmission, further demonstrating the regulatory role of m6A in chemically induced changes in neural plasticity.^61^

Collectively, these findings highlight the indispensable role of m6A methylation in both the development and maintenance of neural plasticity. By regulating synaptic plasticity and neural function, m6A serves as a critical link between environmental stimuli, gene expression, and behavioral outcomes. Further investigation into the mechanisms of m6A in neural plasticity will deepen our understanding of the pathogenesis of mental disorders, such as depression, and provide a strong theoretical foundation for the development of innovative therapeutic strategies.

#### The Role of m6A Methylation in Neurotransmitter System

Studies have highlighted that m6A modification plays a pivotal role in regulating neurotransmitter systems by modulating the expression of key genes involved in neurotransmitter synthesis, release, and receptor signaling. This is particularly evident in the dopamine (DA) and serotonin (5-HT) systems, both of which are closely linked to the pathophysiology of mental disorders.

##### The Impact of m6A Methylation on the DA System

The DA system is crucial for regulating emotions, motivation, and reward-related behaviors.^62^ N6-methyladenosine methylation modulates the function of dopaminergic neurons by primarily affecting the synthesis and release of DA. For example, the deletion of METTL14 disrupts dopaminergic function in the substantia nigra, impairing DA release and activation of associated signaling pathways.^63^ Moreover, small-molecule inhibitors targeting the m6A demethylase FTO have been shown to significantly increase the survival of dopaminergic neurons, indicating that m6A plays a key role in maintaining the integrity of the DA system.^64^

Further research has demonstrated that overexpression of FTO or the inhibition of m6A modification leads to a reduction in m6A levels within the striatum, resulting in decreased DA levels. This alteration in DA signaling may contribute to the pathophysiology of PD, suggesting that m6A could be a potential therapeutic target for DA-related disorders.^65^ Additionally, METTL3, another important methyltransferase, regulates DA synthesis by modulating the expression of DA-related genes, further influencing neural signaling.^66^ These findings underscore the potential of m6A methylation as a therapeutic target in conditions like PD and schizophrenia, where dopaminergic dysfunction plays a central role.

##### The Impact of m6A Methylation on the 5-HT System

Similar to the DA system, the 5-HT system is vital for regulating mood, emotion, and behavior. m6A modification has been shown to influence 5-HT levels and neuronal transmission by regulating the expression of genes involved in 5-HT synthesis and metabolism.^67^ Moreover, emerging evidence suggests that m6A methylation may modulate serotoninergic signaling by affecting the expression of 5-HT transporters (SERT) and various 5-HT receptors.^68^ These findings indicate that m6A methylation is a promising target for addressing 5-HT-related disorders, such as depression.

#### The Impact of m6A Methylation on Neuroinflammatory Responses

Increasing evidence suggests that m6A methylation significantly influences the expression of inflammation-related genes and contributes to the development of various inflammation-associated diseases. For instance, FTO modulates the stability of cGAS mRNA by regulating m6A methylation levels, thereby alleviating neuroinflammation caused by cerebral ischemia-reperfusion.^36^ Moreover, m6A modification plays a critical role in the inflammatory response of microglia. Exosomal FTO derived from neural stem cells protects neurons from inflammatory damage by suppressing the m6A modification of NRF2 mRNA in microglia, thus attenuating neuroinflammatory processes.^69^ Furthermore, METTL3, a key m6A methyltransferase, has been shown to reduce microglial inflammation and decrease blood-brain barrier permeability by modulating m6A levels. This regulation alters the expression of miR-335-3p and inhibits the activation of the NLRP3 inflammasome, a critical player in inflammatory responses.^70^ This mechanism is particularly significant in the context of cerebral ischemic stroke, where it helps protect the brain from further damage.

Given the close correlation between neuroinflammation and the pathogenesis of mental disorders, as well as the role of m6A methylation in regulating neuroinflammatory processes, m6A methylation offers a promising target for the development of novel therapeutic strategies aimed at treating inflammation-related disorders.

## M6A METHYLATION MODIFICATION AND COMMON PSYCHIATRIC DISORDERS

### The Role of m6A Methylation Modification in Depression

Depression is a common psychiatric disorder characterized by persistent low mood, loss of interest or pleasure in activities, accompanied by intense feelings of guilt, changes in sleep and appetite, and other symptoms. In severe cases, cognitive dysfunction, abnormal behavior, and even hallucinations and delusions may occur.^71^^,^^72^ The pathogenesis of depression is complex, resulting from the interplay of social, psychological, and biological factors. According to data from the World Health Organization (WHO), depression is one of the most prevalent psychiatric disorders globally, affecting approximately 5% of adults. This prevalence is even higher in developed countries, where it ranges from 5% to 10%. Further investigation into the mechanisms underlying depression will help identify more effective prevention strategies and therapeutic targets.

Recent studies have indicated that m6A methylation may play a crucial role in the pathogenesis of depression (Table 1). In the PFC brain region of patients with depression, the expressions of METTL16, YTHDC1, and YTHDC2 show cell-specific dysregulation, and this dysregulation exhibits gender-specific expression differences.^73^ The significant changes in m6A modification levels, in turn, affect the expression of genes related to emotional regulation and stress responses.^87^^,^^88^ Similarly, stress stimulation alters the transcriptional landscape of m6A methylation in mice, contributing to the development of depressive-like behaviors.^89^ These findings suggest that m6A methylation may serve as a potential biomarker and therapeutic target for depression.

**Table 1:** Roles and mechanisms of m6A modification in various mental disorders.

**Diseases**	**Tissues**	**m6A regulator**	**Effect**	**Molecular mechanisms**	**References**
**MDD**	PFC (human)	METTL16, YTHDC1, YTHDC2	/	*METTL16*↑, *YTHDC1*↑, *YTHDC2*↑…	^73^
	Hip (mouse)	ALKBH5	Function of astrocytes	circSTAG1↓—ALKBH5 in cytoplasm↓—*Faah* m6A↓—FAAH degradation↓	^74^
	Hip (human/mouse)	FTO	Synaptic plasticity	FTO↓—*Adrb2* m6A↑—ADRB2↓—c-MYC↓—SIRT1↓	^43^
	VTA (mouse)	FTO	Mediate the effect of tricyclic antidepressants	Drug treatment—FTO↑—Cartpt and Ucn m6A↓—the transcription of *Cartpt* and *Ucn*↓	^75^
	Hip (rat)	METTL3	Cognitive impairment of depression	METTL3↑—pri-miR-221 m6A↑—binding with DGCR8↑—miR-221-3p↑—*Gab1*↓	^76^
	PFC, CE, frontal lobe, hypophysis (rat)	METTL3, FTO, YTHDF2 , HNRNPC	Immune response	m6A modification affect differentiation of CD4+ T cells and inflammatory responses	^77^
	Hip (mouse)	FTO	Synapse density and synaptic morphology alterations	FTO↓—CaMKII and cAMP↓—SYN and PSD95↓	^42^
	ACC (mouse)	FTO	/	FTO↓—*Mmp-9* m6A↑—proBDNF/mBDNF↑	^78^
**SCZ**	Brain，plasma^*1^ (human)	ALKBH5	Synaptic plasticity of 5-HT neuron	ERVWE1↑—ALKBH5↑—*HTR1B* m6A↓—HTR1B↑— p-ERK, p-ELK1, Arc↑	^68^
	Blood^*2^ (human)	FTO	The occurrence of metabolic syndrome	Correlation between FTO rs9939609 and metabolic syndrome in chronic schizophrenic patients treated with AP	^79^
**ASD**	Plasma^*3^ (human)	/	/	There are significant alterations in the m6A modification levels of genes related to immune responses and neural development.	^80^
	STR (mouse)	YTHDF1	Synapse formation and synapse function	YTHDF1↓— degradation of m6A-modified *eIF4E*↓—*eIF4E*↑—dysregulation of translation	^81^
	Hip (mouse)	METTL3	Hippocampal neuron apoptosis	METTL3↓—*Malat1* m6A level↓—MALAT1 stability↓—SFRP2↑—β-catenin↓	^82^
**OUD**	Hip (mouse)	FTO	Synaptic maturation and localization	FTO↓—m6A level↑—dysregulation of m6A peaks in mRNA and lncRNA	^83^
	STR (mouse)	METTL14	Oxycodone-induced locomotor activity changes	METTL14↑—m6A level↑—IncRNA HOTAIR↑—LSD1↑—H3K4me1↓—PP1α↓	^84^
	Primary cortical cultures (rat)	ALKBH5	Cell signaling cascades, cytostructural components and inflammation	ALKBH5↓—differential methalation of mRNAs and non-coding RNAs	^85^
	NAc (human)		Alcohol-induced neuroadaptative changes	m6A methylomic profiling of coding and non-coding RNAs	^86^

Plasma or blood-derived population: *1: Patients with the first episode of schizophrenia (*n* = 44); Healthy control group (*n* = 37). *2: Patients with schizophrenia who have received antipsychotic medication treatment for at least 1 year in the Malaysian region (*n* = 206). *3: There were 75 pairs of twins. One of them had autism, while the other did not (age: 4-18). Abbreviations: ACC, anterior cingulate cortex; ASD, autism spectrum disorder; CE, cerebellum; DSC, dorsal spinal cord; Hip, hippocampus; MDD, major depressive disorder; NAc, nucleus accumbens; OUD, opioid use disorder; PFC, prefrontal cortex; SCZ, schizophrenia; STR, striatum; VTA, ventral tegmental area; ↑, for upregulation; ↓, for downregulation.

Research on the Han Chinese population has revealed that single nucleotide polymorphisms in the demethylase ALKBH5 may be risk factors for MDD in this group.^41^ In a chronic unpredictable stress (CUS) mouse model, circSTAG1, a circular RNA identified from the hippocampus, was found to block the nuclear translocation of ALKBH5, promoting high methylation of fatty acid amide hydrolase (FAAH) mRNA in astrocytes, thereby reducing FAAH degradation and preventing astrocytic damage, which alleviates depressive-like behavior. This suggests that circSTAG1-mediated m6A methylation could become a new target for depression treatment.^74^

FTO, another demethylase, is significantly downregulated in the hippocampus of both MDD patients and depressive mouse, leading to elevated methylation levels of Adrb2 mRNA. This methylation change mediates the development of depressive-like behaviors via the ADRB2-c-MYC-SIRT1 signaling pathway, which is also closely related to the therapeutic effects of the antidepressant fluoxetine.^43^ In parallel, studies have demonstrated that FTO expression in the ventral tegmental area (VTA) contributes to the efficacy of tricyclic antidepressants (TCAs), further supporting its relevance in antidepressant response.^75^ Moreover, in a chronic restraint stress model, impaired FTO function in the hippocampus disrupts synaptic plasticity by interfering with the CaMKII/p-CREB signaling cascade and reducing the expression of key synaptic proteins. Pharmacological activation of FTO in this region effectively reverses these deficits, highlighting the FTO-CaMKII/CREB axis as a critical modulator of stress-induced synaptic dysfunction.^42^ Extending beyond depression, downregulated FTO has also been implicated in the pathophysiology of mood disorders comorbid with neuropathic pain (NP). In the chronic constriction injury (CCI) model, reduced FTO expression in the anterior cingulate cortex (ACC) increases m6A methylation of *Mmp-9*, thereby lowering MMP-9 protein levels and disrupting the balance between proBDNF and mature BDNF. These changes collectively contribute to the emergence of anxiety- and depression-like behaviors.^78^

The m6A methyltransferase METTL3 is highly expressed in CUS-induced depressive rat models, and silencing METTL3 improves cognitive deficits. Specifically, METTL3 mediates the m6A modification of pri-miR-221, promoting its maturation and upregulating miR-221-3p, which inhibits *Gab1* expression and exacerbates cognitive deficits related to depression.^76^ Another bioinformatic analysis study found that in depression rat models, genes such as *Mettl3*, *Fto*, *Ythdf2*, and *Hnrnpc* are significantly upregulated, with 66.7% of these genes being core genes regulated by m6A modification, highlighting their crucial role in the onset and progression of depression.^77^

As an important RNA modification, m6A methylation has shown potential as a biomarker in the diagnosis of various diseases, including cancers,^90^ neurological disorders,^91^ and cardiovascular diseases.^92^ Regretfully, despite recent advances in understanding the role and mechanisms of m6A methylation in the pathogenesis of depression, its application in clinical diagnosis remains limited. To date, few studies have successfully translated m6A-related findings into practical diagnostic tools for MDD. Moving forward, a promising direction lies in profiling the expression of m6A regulatory enzymes and methylation dynamics of m6A in depression-associated risk genes or any novel candidate molecules using liquid biopsy samples of patients. The integration of these epitranscriptomic biomarkers with artificial intelligence-driven analytical frameworks may pave the way for the development of accurate, minimally invasive diagnostic models of depression, representing a critical step toward precision psychiatry.

Preclinical studies have demonstrated that modulating m6A levels can effectively reverse depressive-like behaviors in animal models. For instance, injecting cycloleucine, a compound that reduces m6A methylation by competitively inhibiting methionine adenosyltransferase (the enzyme responsible for synthesizing S-adenosyl-L-methionine (SAM), the primary methyl donor for DNA and RNA methylation) into a chronic social defeat stress (CSDS) mouse model significantly alleviated depressive-like behaviors.^75^ Moreover, targeting m6A regulators to indirectly modulate m6A levels or its recognition has also emerged as a promising strategy for alleviating depressive symptoms.^93^ Despite these encouraging findings, the clinical application of m6A-based therapeutics remains in its early stages. Key challenges include the identification of reliable m6A-modified target genes, the development of selective and safe modulators of m6A machinery, and the establishment of rapid methylation detection methods for clinical use. Furthermore, investigating the combined application of m6A-targeted agents with conventional antidepressants may offer a promising approach to enhance treatment efficacy and mitigate adverse effects.

### The Role of m6A Methylation Modification in Schizophrenia

Schizophrenia is a severe mental disorder characterized by significant geographical and demographic variations. The incidence rate typically ranges from 0.5% to 1% in various countries and regions, although it may be higher in specific populations.^94^ The clinical manifestations of schizophrenia include hallucinations, delusions, disorganized thinking, flattened affect, and cognitive dysfunction. These symptoms severely impair patients’ daily functioning and quality of life.^95^ In recent years, there has been a growing focus on exploring the biological mechanisms underlying schizophrenia, particularly the regulation of gene expression. Among these, m6A methylation modification, as an important epigenetic mechanism, has garnered widespread attention (Table 1).

Research has demonstrated that dynamic changes in m6A modification are crucial for regulating neuronal function and are closely associated with the pathological mechanisms of schizophrenia. In the brain tissue of schizophrenia patients, abnormal levels of m6A modification may lead to dysregulated expression of genes involved in neurodevelopment and neuronal function, which subsequently affect neuronal morphology and functionality, potentially contributing to schizophrenia’s onset.^10^^,^^96^ Notably, maternal immune activation, a well-established risk factor for schizophrenia, is thought to play a key role in the pathogenesis of the disorder, and studies also suggest that m6A modification is involved in this process.^97^

The development of schizophrenia is often associated with disrupted synaptic plasticity.^96^ Studies have shown that the human endogenous retrovirus W family envelope (ERVWE1), an important risk factor for schizophrenia, modulates ALKBH5-dependent m6A modification through the HTR1B-ERK-ELK1-Arc signaling pathway, leading to impaired plasticity of 5-HT neurons.^68^ This mechanism is particularly evident in patients with first-episode schizophrenia. Interestingly, a humanized IgG4 monoclonal antibody, GNbAC1, can antagonize the surface domain of the ERVWE1 protein, and this monoclonal antibody is currently in phase II clinical trials for the treatment of multiple sclerosis. In the future, this monoclonal antibody may potentially serve as a novel approach for treating schizophrenia.^68^

In addition to its role in disease onset, m6A modification is also implicated in the response to antipsychotic treatments. Studies have found significant changes in the methylation patterns of both DNA and RNA in schizophrenia patients following conventional drug treatment.^68^ These findings suggest that m6A modification may play a critical role in the drug response, offering new possibilities for monitoring and evaluating treatment efficacy in the future. Notably, individuals with schizophrenia exhibit a markedly higher incidence of metabolic syndrome compared to the general population. Genetic studies have further revealed that polymorphisms in the *FTO* gene are associated with increased susceptibility to metabolic syndrome and may contribute to the pathogenesis of schizophrenia, thereby offering a possible predictive marker for the disorder.^79^^,^^98^

Through the analysis of m6A methylation patterns, researchers are gaining a deeper understanding of the biological basis of schizophrenia, thereby providing new approaches for early screening and personalized treatment.

### The Role of m6A Methylation Modification in ASD

Autism spectrum disorder is a neurodevelopmental disorder primarily characterized by impairments in social interaction, communication skills, along with repetitive behaviors and restricted interests.^99^^,^^100^ According to the fifth edition of the Diagnostic and Statistical Manual of Mental Disorders, Fifth Edition (DSM-5) by the American Psychiatric Association, the symptoms of ASD typically appear before the age of 3 and exhibit considerable heterogeneity among individuals. Epidemiological studies indicate that the prevalence of ASD has risen significantly over recent decades, with current estimates suggesting that 1 in 68 children is diagnosed with the disorder.^101^ This increase may be due to a variety of factors, including broader diagnostic criteria, environmental influences, and genetic predispositions. The pathogenesis of ASD is highly complex, involving interactions between genetic, environmental, and biological factors, which underscores the importance of further research into its underlying mechanisms.^102^

Integrated analysis of multi-omics data has revealed that abnormalities in m6A modification are closely linked to the onset of ASD,^103^ along with repetitive behaviors and restricted interests (Table 1). According to the Diagnostic and Statistical Manual of Mental Disorders, Fifth Edition (DSM-5), symptoms of ASD typically manifest before the age of 3 and exhibit significant heterogeneity across individuals. Epidemiological studies indicate a significant increase in the prevalence of ASD in recent decades, with current estimates suggesting that 1 in every 68 children is diagnosed with ASD.^101^ This rise may be associated with various factors, including broader diagnostic criteria, environmental influences, and genetic susceptibility. Moreover, the pathogenesis of ASD is extremely complex, involving the interplay of genetic, environmental, and biological factors,^102^ which underscores the importance of further research into its underlying mechanisms.

In recent years, an increasing body of research has suggested that m6A methylation modification may play a crucial role in the pathogenesis of ASD. Through integrative multi-omics analyses, studies have found that abnormal m6A modification is closely associated with the onset of ASD.^103^ For example, transcriptomic sequencing of peripheral blood samples from ASD patients has revealed significant alterations in m6A modification levels of genes related to immune response and neurodevelopment, providing new insights into the molecular mechanisms underlying ASD.^80^ Moreover, changes in m6A modification are associated with key clinical phenotypes of ASD, such as deficits in social skills and the presence of stereotypical behaviors.

Animal models and in vitro cell experiments have also confirmed the involvement of m6A modification in ASD pathogenesis. By manipulating m6A modification levels in cells using gene editing technologies, researchers observed gene expression changes associated with ASD, providing direct evidence for the role of m6A modification in the disorder.^104^

N6-methyladenosine modification contributes to the pathological process of ASD by regulating key signaling pathways. One critical m6A reader protein, YTHDF1, plays an essential role in axon and dendrite development. YTHDF1 regulates synaptic development and plasticity by promoting the translation of Robo3.1, an axon guidance protein. However, when mutations occur at the m6A modification sites, YTHDF1 loses its ability to regulate Robo3.1 translation, resulting in abnormal synaptic transmission and plasticity, which subsequently impairs memory consolidation.^81^ Additionally, studies suggest that YTHDF1 may be associated with the stereotypical behaviors observed in ASD. YTHDF1 interacts with eukaryotic initiation factors (eIFs) and modulates mRNA translation to affect protein synthesis. In ASD patients, variations in the *EIF4E* gene may alter the function of YTHDF1, leading to abnormal mRNA translation and triggering repetitive and rigid behaviors.^105^

Moreover, research has found that in the hippocampal tissue of ASD patients or model mice, the expression of the m6A methyltransferase METTL3 is significantly downregulated, accompanied by downregulation of long non-coding RNA MALAT1 and the Wnt/β-catenin signaling pathway, while the expression of the inhibitory molecule SFRP2 is upregulated. These changes contribute to the apoptosis of hippocampal neurons, exacerbating the pathological process of ASD.^82^ Targeting and regulating METTL3 has been shown to reverse these alterations in signaling pathways and improve autistic-like behavior, suggesting that METTL3 could serve as a potential diagnostic biomarker for ASD and a target for future therapeutic interventions. However, these findings require further validation in larger clinical studies and animal models.

Abnormal m6A modification in ASD patients exhibits significant molecular characteristics, which could potentially serve as biomarkers for the disorder. By detecting m6A levels in peripheral blood, researchers could identify high-risk individuals for ASD before clinical symptoms appear, facilitating early intervention. Additionally, the combined use of m6A detection with other biomarkers, in conjunction with multi-omics data, could significantly enhance the sensitivity and specificity of ASD screening.^106^

Current research indicates that targeting m6A methylation regulation may provide new therapeutic strategies for ASD. For example, modulating METTL3 expression levels could repair the Wnt/β-catenin signaling pathway, reduce hippocampal neuronal apoptosis, and improve ASD-related behaviors. This highlights the important role of m6A modification in the pathogenesis of ASD and its potential as a therapeutic target. Future large-scale clinical studies and in-depth mechanistic investigations will provide further evidence to support this field.

### The Role of m6A Methylation Modification in Drug Addiction

Drug addiction is a chronic and relapsing brain disorder, primarily characterized by compulsive drug seeking and use, along with a loss of control over drug consumption.^107^ Addictive behaviors often manifest as individuals compulsively and persistently using substances to either experience the pleasurable effects or to avoid the discomfort caused by withdrawal symptoms. Such behaviors not only affect an individual’s mental state but also have profound physical consequences.^108^ Common addictive substances include opioids, cocaine, sedatives, and certain prescription drugs. The development of drug addiction involves extensive changes in the structure and function of the nervous system, which promote drug-seeking behaviors and eventually lead to addiction disorders.^109^ From a neurobiological perspective, drug addiction is influenced by an individual’s genetic susceptibility, environmental factors, and behavioral stimuli. This addictive state is often accompanied by physiological imbalances in the brain, leading to intense cravings for the substance, while withdrawal symptoms can cause general discomfort, irritability, lack of concentration, and sleep disturbances.

#### Cocaine Addiction and m6A Modification

Cocaine is a highly addictive stimulant. Research has shown that in a cocaine-induced mouse conditioned place preference (CPP) experiment, the expression of FTO in the hippocampus was significantly downregulated, while the expression of methyltransferases such as METTL3 and METTL14 did not show significant changes. This suggests that FTO may play a key role in the regulation of cocaine addiction. Further analysis through methylated RNA immunoprecipitation sequencing (MeRIP-Seq), a technique that maps m6A-methylated RNA, revealed that the overall m6A modification levels in the hippocampus of cocaine-treated mice were significantly elevated when compared with control group, with changes in m6A peaks primarily involving genes related to glutamatergic synapses, long-term potentiation (LTP), axon guidance, and neuronal function. These findings indicate that the dynamic changes in FTO expression and m6A modification may mediate the onset of cocaine addiction by regulating synaptic maturation and localization.^83^

#### Opioid Addiction and m6A Modification

In a separate study using opioids such as oxycodone to induce rewarding behaviors in mice, researchers observed a marked increase in the protein levels of METTL14 and overall m6A modification in striatal neurons. In contrast, the expression of PP1α, a protein phosphatase, was found to decrease. These results suggest a close relationship between m6A methylation and opioid addiction. Further investigation pointed to lncRNA HOTAIR as a potential mediator in this process.^84^ Through the regulation of specific non-coding RNAs and their downstream targets, m6A methylation may influence neuronal activity and behavioral responses, thereby exacerbating opioid addiction.

#### Morphine Addiction and m6A Methylation

In contrast to cocaine addiction, a study on morphine addiction in mice found that neither acute nor chronic morphine exposure significantly altered m6A modification levels or the expression of m6A methyltransferases (such as METTL3) in the brain. This suggests that m6A methylation modification may not be involved in the regulatory process of morphine addiction, or its mechanisms may differ from those involved in other addictive substances.^110^ However, in another study involving chronic morphine treatment in rats, changes in m6A modification were detected in the PFC and possibly mediated by ALKBH5.^85^ The discrepancies among these findings may be attributed to several factors, including differences in experimental design, drug dosage, duration of exposure, the brain regions analyzed, and the sensitivity of the methodologies employed. Therefore, further evidence is needed to clarify the role of m6A modification in morphine addiction.

#### Alcohol Use Disorder and m6A Modification

Alcohol use disorder (AUD) is another prevalent form of addiction characterized by compulsive drinking, alcohol craving, increased tolerance, and severe withdrawal symptoms. The nucleus accumbens (NAc), a key brain region involved in DA-mediated reward and pleasure, has been implicated in the development of alcohol addiction. In a mouse model of long-term alcohol consumption, RNA methylation changes were associated with specific gene expression alterations, leading to enhanced neural adaptation to alcohol. Through epigenomic microarray analysis, researchers identified 26 mRNAs and 4 lncRNAs in the NAc of AUD subjects that were highly methylated, while the methylation level of 3 mRNAs and 1 lncRNA was significantly reduced. These results suggest that m6A methylation may play a role in the neural adaptation processes underlying alcohol addiction. However, these findings need to be replicated in larger sample sizes, and future research should explore whether these changes are gender- or population-specific.^86^ These methylation alterations could influence the transcription and translation of genes involved in opioid receptor signaling, further regulating the effect of alcohol on the brain^111^ (Table 1).

## CONCLUSION AND FUTURE OUTLOOK

This review focused on 4 major psychiatric disorders—depression, schizophrenia, ASD, and drug addiction. Although other conditions such as anxiety, attention-deficit/hyperactivity disorder, and bipolar disorder also exhibit high prevalence and clinical importance, current research on m6A modification in these disorders remains limited. This gap underscores the need for future investigations, which may contribute to the identification of novel biomarkers and therapeutic targets across a broader range of psychiatric conditions.

Through an integrated analysis of m6A modifications in the aforementioned disorders, we found that several key m6A-regulating enzymes, such as METTL3, ALKBH5, and FTO, exhibit similar patterns of dysregulation across different mental illnesses. Moreover, these enzymes are primarily involved in the onset and progression of psychiatric disorders by modulating similar processes, including neuroinflammation, neurodevelopment, and synaptic plasticity. These parallels not only reflect the presence of shared pathogenic mechanisms among various mental disorders but also underscore the indispensable role of m6A regulatory enzymes in initiating epitranscriptomic alterations that contribute to disease development.

Nevertheless, it is important to recognize that, although current studies on m6A modification in mental disorders remain limited, distinct brain region- or cell types-specific differences have been observed in the patterns of m6A modification and the expression of its regulatory enzymes across different psychiatric conditions. The specific RNAs undergoing m6A modification, as well as the downstream signaling pathways they modulate, also vary widely. Therefore, further elucidation of the downstream molecular networks governed by m6A modification is critically needed.

Looking forward, this complexity poses an important question for therapeutic development: should strategies targeting m6A pathways adopt a broad-spectrum approach, or be tailored specifically to the molecular landscape of each disorder? Addressing this question will be essential for translating m6A-related findings into clinically meaningful interventions.

Furthermore, the emergence of single-cell sequencing technologies provides new opportunities to investigate m6A methylation dynamics at high resolution. Future studies leveraging these approaches will enhance our understanding of m6A functions in distinct neuronal subtypes and disease-relevant cellular contexts.

Finally, the development of small-molecule modulators—such as agonists or antagonists of m6A-associated proteins—holds great promise for therapeutic innovation. Such tools may enable precision medicine approaches tailored to individual molecular profiles. Continued research in this direction will not only deepen our understanding of m6A biology but also advance the design of effective, targeted treatments for psychiatric disorders.

## Data Availability

Not applicable.
